# Editorial: Society, organizations and the brain: building towards a unified cognitive neuroscience perspective, volume II

**DOI:** 10.3389/fnhum.2024.1379995

**Published:** 2024-02-15

**Authors:** Sven Braeutigam, Nick Lee, Carl Senior

**Affiliations:** ^1^Medical Sciences Division, Oxford Centre for Human Brain Activity, University of Oxford, Oxford, United Kingdom; ^2^Warwick Business School, Faculty of Social Sciences, University of Warwick, Coventry, United Kingdom; ^3^School of Psychology, Aston University, Birmingham, United Kingdom

**Keywords:** organization, coaching, neuroscience, gender differences, consumer

“The most corrected copies are commonly the least correct.” This paradoxical saying – attributed to seventeenth century British statesman and master of the English language Francis Bacon – illustrates aptly the situation twenty first century scientists face in their endeavor to unravel the complex mechanisms underlying the inner workings of organizations and societies, and more generally, the interactions between humans and between humans and the environment. Undeniably, the amount and variety of empirical data as well as theoretical approaches seems bewildering, conflicting, inexplicable, and even illogical at times, which poses challenges that need to be overcome to unearth structures, mechanisms, and, ultimately, meaning.

Since the publication of Volume I of this Research Topic 8 years ago, undeniably, our world has become even more complex, where, among others, a pandemic, demographic changes, and a gradual reshape of the power balance between east and west constitute significant factors, the impact of which percolates down through the individual. Notwithstanding, a group of authors from the first volume and new contributors have taken up the challenge providing further thoughts on mechanisms relevant at the societal level.

Drawing on existing evidence from neuroscience, Rippon argues that it is insufficient to consider only endogenous, brain-based explanations of the gender-related differences observed in society and organization. In addition one should take into account research demonstrating the behavioral consequences and cortical manifestations of social experiences, specifically negative ones. As a test case, the author highlights the so-called gender equality paradox, which refers to the finding that male over-representation in the sciences correlates with the level of gender equality in a society, apparently contradicting the common assumption that reducing the gender equality gaps should result in increasing numbers of women in science (Stoet and Geary, 2012; Williams and Ceci, 2015).

Jack et al. posit that effective coaching, which can yield improved personal development, must consider the different aspects of a client's self, instead of focusing exclusively on their Ideal self. In support of their claim, the authors provide fMRI data suggesting that the presumed conflict between Ideal and Real self is, at the neuronal level, related to an attention conflict generated by stimuli that are in favor of either global or local perceptual features. The author's findings might point to an explanation of the so-called Gestalt's Paradox referring to the observation that people are more likely to change in the future the more they accept themselves as they are now (Kirchner, 2000).

In an opinion paper, Hoffmann et al. suggest that coaching could both inspire and inform neuroimaging studies of brain mechanisms involved in understanding speech that drives complex social behaviors, an issue that has been given relatively scant attention so far.

The remaining papers address consumer neuroscience, an area of research that is gaining momentum globally. Cayolla et al. studied the neuronal correlates of fandom. Their fMRI data suggests that loyal fans of weak football teams activate more strongly neuronal circuitry associated with attention and the integration of visual-spatial information compared to fans of strong teams. These results might help to explain the paradoxical observation that fans of poorly performing teams often exhibit strong fan identity and are tightly “fused” to their clubs, which appears at odds with a general behavior know as loss aversion (Newson et al., 2023).

Gier et al. study the neuronal correlates of message framing. Their fMRI data show a specific cortical activation (insula) evoked by negative frames for objects (message targets) that carry a negative connotation, but not for positive objects. This could lead to a useful biomarker to study the intent-purchase gap (e.g., Carrington et al., 2014), which refers to the observation that consumers' intentions (as measured through surveys) often do not or only incompletely match actual purchase decisions (measured through sales data).

Foxall offers a deeper look at a class of theoretical approaches to consumer intention and choice known as behavioral perspective models. Drawing on known functional neuroanatomy, the author propose a model extension that avoids a bipolar treatment of the automatic and controlled aspects of consumer behavior and could lead to better understanding of how (everyday) routine choice might turn into extreme choice (see also Roy and Datta, 2022).

Finally, Haidinger and Koller provide a brief overview of consumer neuroscience, with an emphasis on areas where it can contribute insights beyond conventional methods. Specifically, the authors call for more research addressing the advantages, challenges, and ethical concerns related to consumer neuroscience (see also Braeutigam and Kenning, 2022).

The editors are satisfied that Volume II of this Research Topic has advanced the debate, where big and complex issues relevant at economic, organizational, and societal levels can be approached from a neuroscience perspective. Reassuringly, experimental designs become increasingly “real-world” like, going far beyond the simplistic, abstract stimulus-based approaches often found in neuroimaging studies. In addition, theories are being refined and researchers are increasingly think in multi-polar and translational terms, where approaches might become truly interdisciplinary, a key issue already highlighted in the first volume. Such advances, however, cannot shadow the fact that we are still far away from a genuinely unified cognitive neuroscience perspective that could consistently explain the complex web of societies and organizations. Perhaps there is a true paradox out there which might be impossible or difficult to explain in a holistic, all-encompassing fashion.

## Author contributions

SB: Writing—original draft, Writing—review & editing. NL: Writing—review & editing. CS: Writing—review & editing.
